# Engineering Auronidin Production in *Marchantia polymorpha* Enables a New Class of Plant-Derived Textile Pigments

**DOI:** 10.1021/acssynbio.6c00118

**Published:** 2026-07-18

**Authors:** Aurore Lormet, Jim Haseloff, Facundo Romani

**Affiliations:** Department of Plant Sciences, 2152 University of Cambridge, Cambridge CB2 3EA, U.K.

**Keywords:** auronidin, *Marchantia polymorpha*, textile dyes, MYB14, synthetic biology, sustainable biotechnology

## Abstract

Plant-derived pigments offer sustainable alternatives to synthetic colorants, yet their practical deployment in textiles is limited by restricted chemical diversity and low abundance. Liverworts represent a source of diverse chemical compounds, and the model liverworts *Marchantia polymorpha* are emerging as chassis for bioengineering and synthetic biology. Here, we report the biotechnological application of auronidins, a rare class of flavonoid pigments, as textile dyes. Using *M. polymorpha*, we engineered enhanced auronidin production through controlled expression of the R2R3-MYB transcription factor Mp*MYB14*. We systematically benchmarked constitutive and inducible gene expression systems, including heat-shock, glucocorticoid receptor, and β-estradiol (XVE) circuits, identifying inducible strategies that decouple biomass accumulation from secondary metabolite production while achieving high pigment yields. Extracted auronidins were used to dye cotton yarn directly, demonstrating the feasibility of auronidins for textile dyeing. Our results establish *M. polymorpha* as a versatile plant chassis for programmable secondary metabolite production and introduce auronidins as a promising natural pigment platform for sustainable textile biotechnology.

## Graphical abstract

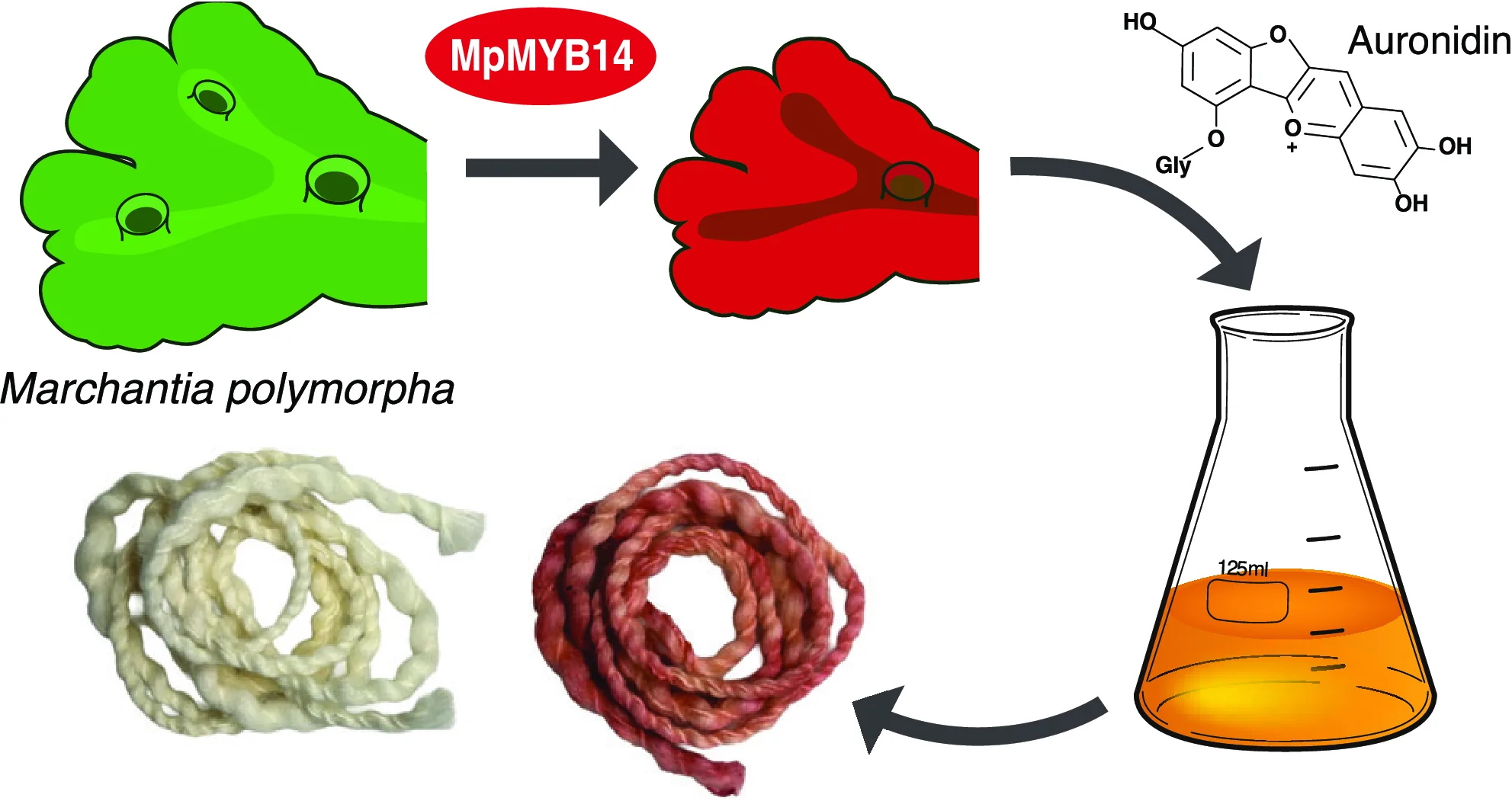

Plant pigments are attracting attention as sustainable alternatives to synthetic colorants for foods, cosmetics, and advanced materials. Besides their vivid colors, many plant-derived pigments offer antioxidant, antimicrobial, and anti-inflammatory activities that add value in functional foods and materials.^1^ However, the palette of plant-derived pigments traditionally used for textile dyeing is chemically and chromatically constrained, being dominated by a limited number of compound classes, such as anthraquinones, flavonoids, carotenoids, and indigoids. While these pigments have enabled natural dyeing practices for centuries, they occupy a restricted color space and often display suboptimal stability or strong dependence on mordants and processing conditions. Moreover, many naturally occurring dyes accumulate at low concentrations or in highly specialized tissues, necessitating large-scale cultivation and extraction to achieve industrially relevant yields. These limitations represent a major bottleneck for the broader adoption of sustainable plant-based dyes.

Plant metabolic engineering provides an efficient route to high levels of natural products with enhanced functional properties. Recent advances in crop engineering for pigment production show promise for sustainable textile applications.^2^ A biotechnological approach offers a route to overcoming bottlenecks by enabling the controlled production of alternative pigment chemistries in engineered plant systems.

Liverwort species produce riccionidin A, a unique type of red pigment that belongs to the auronidin class.^3^ Notably, liverwort auronidins are predominantly associated with the cell wall rather than being vacuole-localized, distinguishing them from many commonly studied plant pigments.^4^ In the model liverwort *Marchantia polymorpha*, the biosynthesis of auronidins is induced under environmental stress and nutrient deprivation and serves roles in photoprotection and pathogen resistance.^5,6^ This process is controlled by the R2R3-MYB transcription factor Mp*MYB14*, which is required and sufficient to activate endogenous biosynthetic genes.^6,7^

*M. polymorpha* is an emergent chassis for plant synthetic biology.^8^ It has already been used for the production of heterologous proteins^9,10^ and secondary metabolites^9,11,12^ at a proof-of-concept level. A remaining challenge is the deployment of endogenous compounds with immediate functional applications that cannot readily be accessed in a conventional chassis. Auronidins are a lineage-specific innovation of liverworts not accessible in conventional microbial or plant chassis. They present several properties that make them attractive candidates for textile dyeing. Unlike most flavonoid pigments, auronidins exhibit intrinsic fluorescence, a distinctive optical property with potential for functional textile applications, and their color range is underrepresented among commonly used natural dyes. Here, we engineer plants with increased auronidin accumulation via MpMYB14 expression and report the first application of this class of natural products for textile dyeing. Together, these results highlight *M. polymorpha* as a promising biological chassis for metabolic engineering and auronidins as a distinct natural pigment for textile applications.

Auronidins derive from the flavonoid pathway (Figure 1A), but the precise metabolic pathway for their biosynthesis has not been elucidated yet.^3,13^ It has been previously shown that the transcription factor Mp*MYB14* is necessary and sufficient to induce the expression of enzymes associated with its biosynthesis and increase auronidin biosynthesis.^6,7^ In plants with high accumulation of auronidins (_pro_ *35S:*Mp*MYB14)*, analysis of visible absorption spectra of methanol extract from overexpressing plants showed an absorbance peak characteristic of auronidins at 490 nm, which has considerably lower intensity in the wild-type control extract and is absent in extracts of the Mp*myb14* loss-of-function mutant (Figure 1B). The peak coincides with the maximum absorption of the 4-neohesperidoside glycoside of the auronidin riccionidin A.^3^ When excited at 490 nm, the extract displays a specific emission spectrum maximum at 570 nm (Figure 1C). While absolute auronidin concentrations could not be determined due to the lack of purified standards, fluorescence (Ex 490 nm; Em 570 nm) provides a robust and specific comparative metric for subsequent analysis (Supplemental Figure 1).

**Figure 1:**
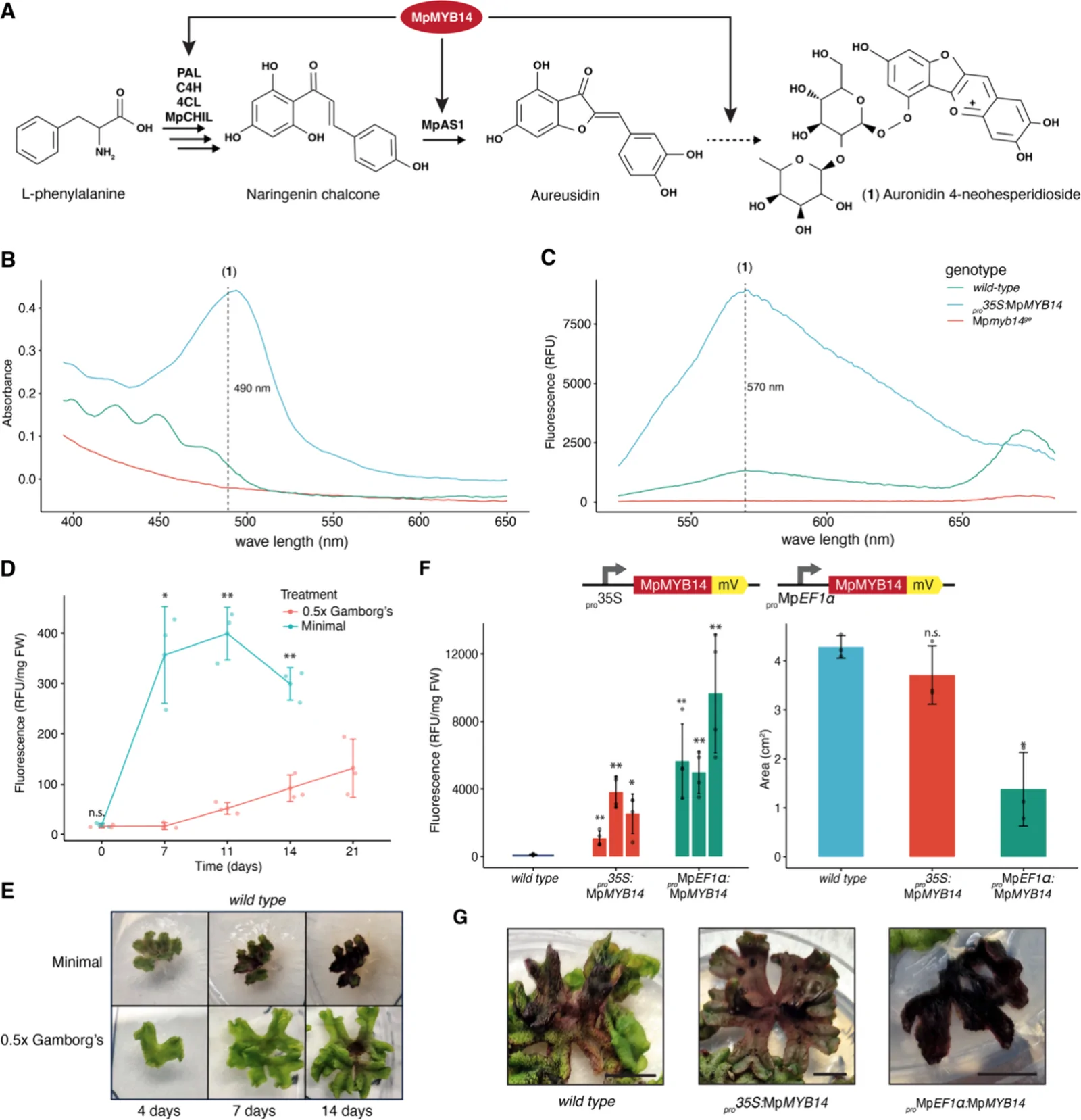
Biosynthesis of auronidins in *Marchantia polymorpha*. (A) Current pathway for auronidin biosynthesis in *M. polymorpha*. The dashed arrow indicates the reaction steps for which enzymes have not yet been identified. MpAS1, aureusidin synthase; MpCHIL, chalcone isomerase-like; PAL, phenylalanine ammonia-lyase; C4H, cinnamate 4-hydroxylase; 4CL, 4-coumarate:CoA ligase. Absorbance (B) and the fluorescence (C) spectra of methanol extracts from WT, myb14 mutant, and pro35S:MpMYB14 M. *polymorpha* lines. The absorbance peak corresponding to 4-neohesperidoside auronidin (**1**) is highlighted as the main peak in the constitutive line (Absλ = 490 nm) and the corresponding emission peak (Emλ = 570 nm). (D) Time course of auronidin quantification in wild-type *M. polymorpha*. Gemmalings were grown for 10 days under complete media (0.5× Gamborg’s B5 media) and either transferred to starvation conditions (1% sucrose (w/v) media) or maintained in complete media. Quantification of auronidins as accumulation of relative fluorescence units per milligram of fresh weight (RFU/mg FW) over time. (E) Representative pictures of plants under starvation (top) or control (bottom) at days 0, 4, 7, and 14. (F) Left, quantification of auronidins in 3-week-old *M. polymorpha* gemmalings overexpressing MpMYB14 fused to mVenus (mV) under _pro_35S, wild type control, and sporelings of equivalent developmental time under _pro_MpEF1α. Three independent transgenic lines are shown. Right, thallus exposed area of 2-weeks-old sporelings from primary transformants. Sporelings were used as _pro_MpEF1α:MpMYB14 plants are unable to produce viable gemma. (G) Representative pictures of transgenic lines and wild type control. Scale bars correspond to 1 cm. Error bars indicate mean values ± SD of at least three biological replicates. Individual values are shown. Significant differences were tested using pairwise *t* test comparing each time to the corresponding control and corrected by Bonferroni (*q-value of <0.05, **q-value of <0.01).

Under standard *in vitro* culture conditions, auronidins are almost undetectable, with concentration estimated at around 14–30 RFU/mg FW (relative fluorescent units per milligrams of fresh weight). In stress, and particular nitrogen starvation conditions, plants accumulate auronidins in higher quantities.^14^ To monitor the dynamics of auronidin accumulation, we grew wild-type plants for 10 days before being transferred to either stress-inducing minimal media containing only 1% sucrose or complete media (0.5× Gamborg’s B5), as described before.^6^ In minimal media, auronidin concentration peaks at ∼400 RFU/mg FW after 11 days. Over time, adult plants on the complete medium also began to accumulate pigment in the center of the thallus but in relatively lower quantities (Figure 1D). This was accompanied by strong growth defects in minimal media, consistent with previous reports.^3^ This provides an initial point to compare auronidin bioproduction and emphasizes the importance of engineering strategies to increase production and mitigating growth penalties.

Increasing the levels of MpMYB14 represents a logical approach for engineering auronidins bioproduction. First, to benchmark different constitutive promoters and compare with natural production, we generated transgenic lines overexpressing Mp*MYB14* under the _pro_ *35S* and _pro_Mp*EF1α*, two commonly used strong promoters for *M. polymorpha*.^9^ As expected, _pro_Mp*EF1α:*Mp*MYB14* lines achieved the highest levels of auronidin fluorescence (∼9000 RFU/mg FW), followed by _pro_ *35S:*Mp*MYB14* lines (∼4,000 RFU/mg FW). These values represent approximately 450-fold and 200-fold increases relative to unstressed wild type and 22-fold and 10-fold increases compared to the highest auronidin concentrations observed in nitrogen-stressed wild-type plants.

_pro_Mp*EF1α* plants exhibited uniform, intense red pigmentation across the thallus, whereas _pro_35S plants showed concentrated red coloration in the thallus center with greener thallus margins (Figure 1E). The high auronidin levels in _pro_Mp*EF1α* lines was accompanied by a significantly smaller plant compared to wild type. On the other hand, the _pro_35S lines displayed a modest size reduction compared to wild type (Figure 1E). This trade-off between bioproduction and growth when transgenes are under different promoters was also described previously.^9^

To bypass the trade-off, inducible systems have been developed that allow tunable expression and decoupling the production of recombinant products from plant growth.^15^ In *M. polymorpha*, the heat-shock inducible promoter _pro_Mp*HSP17.8A1* has been used to produce protein levels of betanins.^9^ Alternatively, steroid and estrogen inducible systems are also compatible with *M. polymorpha*.^16,17^ The first uses a glucocorticoid receptor (GR) which controls nuclear-cytoplasmic localization of a transcription factor, while the second uses a synthetic transcription factor that depends on the presence of β-estradiol to trigger the transcriptional activation of the gene of interest (Figure 2A).

**Figure 2:**
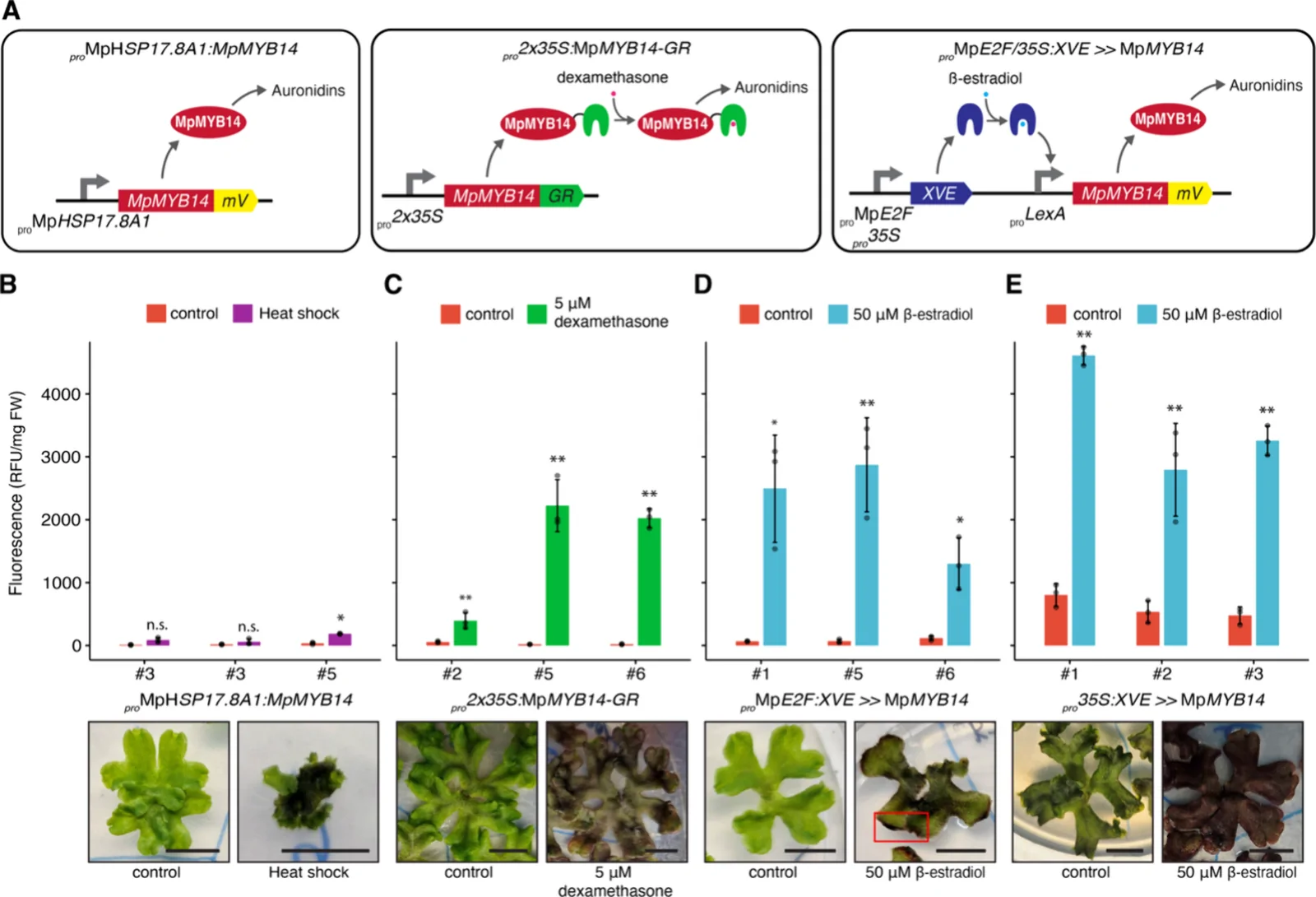
Inducible expression of auronidin. (A) Design of gene construct for auronidin expression. (B–E) Quantification of auronidins in *M. polymorpha* gemmalings from inducible lines (_pro_MpHSP17.8A1:MpMYB14-mV, _pro_35S × 2:MpMYB14-GR, _pro_35S:XVE ≫ MpMYB14-mV, _pro_MpE2F:XVE ≫ MpMYB14-mV). GR: glucocorticoid receptor. mV: mVenus. Plants were grown for 10 days in standard conditions and then exposed to two rounds of heat-shock (37 °C) for 2 h per day or transferred to inducible media as indicated in each plot. Control plants were either not exposed to heat-shock or transferred to media without inducer present. Auronidin levels were quantified after 14 days. Three biological replicates and three independent transgenic lines were used. Bottom, representative pictures of transgenic lines and their respective control. The red box indicates the pigmented apical notch of induced plants. Scale bars correspond to 1 cm. Error bars indicate mean values ± SD of at least three biological replicates. Individual values are shown. Significant differences were tested using pairwise *t* test comparing each time to the corresponding control and corrected by Bonferroni (*q-value of <0.05, **q-value of <0.01).

To compare different inducible strategy, we designed different combinations of genetic parts driving the inducible expression of MpMYB14 (Figure 2A). The transformed plants were initially grown under standard conditions for 10 days before being transferred to inductive conditions for 14 days and harvested. First, with _pro_MpHSP17.8A1 induction for 2 h at 37 °C, we observed a modest increase in auronidin production and strong growth penalty, likely due to heat-induced stress (Figure 2B, Supplemental Figure 2). This highlights the limitations of transient induction for sustained metabolite accumulation, compared with continuous chemical induction via hormone-responsive systems. For the GR construct, we used the strongest promoter available 2x35S^9^ and observed a strong increase of up to ∼2000 RFU/mg FW (Figure 2C). Alternatively, the _*pro*_Mp*E2F:XVE* ≫ Mp*MYB14* synthetic circuit produced up to ∼2800 RFU/mg FW, suggesting that the XVE system is a powerful tool to drive high levels of expression with tight regulation (Figure 2D). This is particularly due to the amplification step of the XVE system (Figure 2A). Yet, the _*pro*_Mp*E2F* is a weak promoter compared to others and is only active in dividing cells.^18^ To obtain even higher levels, we built an alternative version replacing _*pro*_Mp*E2F* with _*pro*_ *35S*. Overall, _*pro*_ *35S:XVE* ≫ Mp*MYB14* resulted in even higher yields, with up to ∼5000 RFU/mg FW but also showed significant activity under noninduced conditions (Figure 2E). Overall, chemical induction of Mp*MYB14* could achieve yields comparable with the best constitutive promoters. Under our regimen, GR and _*pro*_Mp*E2F:XVE* did not compromise the initial vegetative growth phase under the conditions tested (Supplemental Figure 2).

To assess the potential of engineered auronidin production for direct applications, we scaled up the _*pro*_ *35S:*Mp*MYB14* transgenic line to obtain higher biomass by growing plants in hydroponics for 3 weeks. This line was selected for scale-up, as it provides constitutive production without the need for chemical induction, simplifying cultivation in large hydroponic cultures. The resulting extract was filtered and directly used to dye cotton yarn using an exhaustion dyeing protocol at 30% weight of fiber, without the use of mordants.

After dyeing, the cotton yarn exhibited a robust pink coloration under natural light compared with yarn dyed using wild-type extracts (Figure 3A,B). In addition, dyed yarn retained strong auronidin-associated fluorescence (Figure 3B). We next evaluated the effect of commercially available premordant treatments and tested wash fastness. Yarn pretreated with tannins displayed increased color intensity and improved retention after washing compared with unmordanted controls (Figure 3C). Yarn pretreated with tannin and aluminum acetate showed a further increase in color intensity and minimal visible color loss after washing in 50 °C water. Together, these results provide initial evidence that auronidins from engineered *M. polymorpha* can be applied as functional textile pigments, with color depth, fluorescence and reasonable fastness properties within the range observed for commonly used natural dyes.

**Figure 3:**
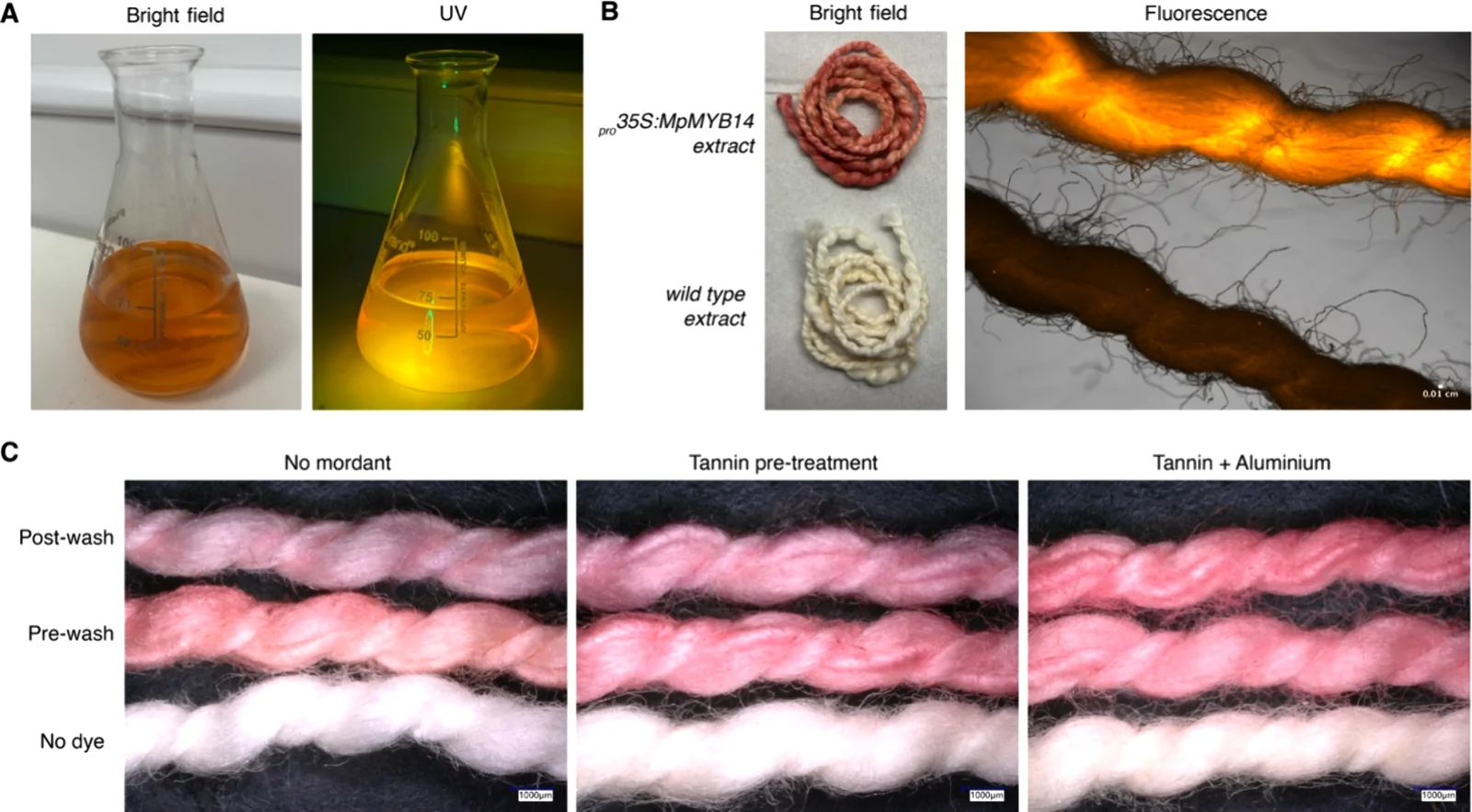
Auronidin extract as textile dyes. (A) A methanol extract from a scaled up culture of _pro_35S:MYB14 thallus in bright light conditions and under 365 nm UV light with a ∼400 nm bandpass filter. (B) Cotton fibers dyed with auronidin pigment extract from *M. polymorpha*_pro_35S:MYB14 line or wild-type plants. Microscopy images under the bright field or fluorescence images of the same fibers under a YFP band-pass filter (500 ± 20 nm excitation and 535 ± 20 nm emission) and colored using the Orange Hot LUT. (C) Representative images of dyed yarn without mordants or pretreated with tannins (gallnut) or aluminum acetate. Scale bars are shown in the respective pictures.

Here, we exploited *M. polymorpha* to engineer substantially elevated levels of auronidins. Compared to the wild-type strain, we observed up to >100-fold higher auronidin yields. MpMYB14 overexpression functions as an effective lever to upregulate native biosynthetic genes and increase auronidin production. These features, together with the capacity for the simple cultivation and fast growth of *Marchantia* species, highlight its wider potential as a green biofactory for secondary metabolism. As shown before, constitutive bioproduction is accompanied by a trade-off in growth.^9^ Here, we further compare the XVE and GR-inducible circuits that have the capacity to mitigate fitness costs by temporally separating biomass accumulation and secondary metabolism. The XVE system under the _*pro*_Mp*E2F* exhibited the best dynamic range among the tested conditions, and the new _*pro*_ *35S* version can provide higher yields but with leaky expression.

Auronidins have not previously been explored as textile dyes, and our dyeing experiments establish a direct biotechnological application for this class of plant pigments. By introducing auronidins as functional textile pigments, this work expands the repertoire of plant-derived dyes by adding a pigment class not previously exploited. In addition to textile dyeing, their intrinsic properties (including fluorescence, UV absorption, and reported antimicrobial activity) may be relevant for further expanding their range of applications.

Recent work has shown that auronidins can undergo polymerization and accumulate as insoluble complexes associated with plant cell walls,^4^ revealing a level of chemical complexity that distinguishes them from many commonly used natural dyes. This could be a major advantage for the pigment retention in textile dyes. However, it is important to note that the major component of the extracts corresponds to soluble glycosylated auronidin, while the polymerized aglycone form entrapped in the cell wall is retained in the insoluble fraction and difficult to extract using conventional methods.^4^ This is a key variable for future optimization, both reducing the polymerization *in planta* to increase extractability and exploiting the polymerization capacity to entangle the pigment within the textile fibers, potentially improving wash fastness.

The bioproduction of auronidins is a powerful proof-of-concept demonstrating the unique advantage of producing endogenous chemicals that cannot be readily produced in conventional chassis. Beyond *M. polymorpha*, liverworts possess a rich biochemical diversity that can be unlocked. The model liverwort offers a closely related chassis with shared precursors and metabolism, which has the potential to enhance the accessibility of liverwort-specific metabolites. A remarkable example is perrottetinene, a psychoactive cannabinoid-like compound present in the leafy liverwort *Radula marginata*,^19^ along with other bis-bibenzyls and terpenoids with potential medicinal applications.^20^ Together, these results position *M. polymorpha* as a versatile platform for translating lineage-specific plant chemistry into functional applications.

## Methods

### Plant Materials and Growth Conditions

*Marchantia polymorpha subs. rudelaris* accessions Cam-1 (male) as the wild-type and Cam-1 × Cam-2 spores for transformation were used. The Mp*myb14* knockout in the Cam background was described in Kongsted et al. (2025). Under standard conditions, lines were grown on complete media containing 0.5× Gamborg B5 medium with 1.2% (w/v) agar micropropagation grade (Phytotech #A296) at pH 5.5 and grown under continuous light at 21 °C with a light intensity of 150 μmol m^–2^ s^–1^.

For nutrient deprivation experiments (9 plants per plate/biological replicate; 3 biological replicates per treatment), gemmae were plated on 0.5× Gamborg media, as described before (complete media) and grown for 10 days under standard growing conditions. After that, for starvation treatment,^6^ plants were transferred to minimal media containing 1% (w/v) sucrose, 1.2% (w/v) agar at pH 5.8 and further grown under standard conditions. Alternatively, control plants were transferred to complete media again.

For experiments with inducible expression (three gemmae per line; three lines per independent transformant), gemmae were plated on complete media and grown under standard conditions for 10 days. After this time, transformants were transferred to complete media without supplementes or supplemented with 5 μM of dexamethasone (#265005, Sigma-Aldrich) or 50 μM of 17-β-estradiol (#E8875, Sigma-Aldrich). Plants were grown on inducible media for 14 days before harvest. Similarly, transformed plants with the heat shock promoter were grown for 10 days in standard conditions and placed in a 37 °C incubator without light for 2h a day for 2 days before being moved back to the standard growth conditions for 1 week.

For scaling up, the *M. polymorpha* line plants were placed in hydroponic vessels allowing a continuous flow of nutrient film solution: 12 mL of each of Samurai Hydro Grow Nutrient A and B (Shogun fertilizers) in 4 L of tap water. The pieces of meristems cut from plants were anchored on a “mat” and covered with a propagator lid, creating a humid environment, over which an LED panel was mounted for optimal growth. Each line was grown for 3 weeks before induction and extraction of auronidin.

### Plasmid Assembly

Constructs were obtained from the one-step GoldenGate cloning protocol for gene cassette described in ref^9^ using the pBy10 and pBy23 acceptors.^21^ L0 parts from the OpenPlant Toolkit^8^ were used as specified in Supplemental Table 1. The coding sequence of MpMYB14 (Mp5g19050) CDS12 part is described in ref 22. The presence of the correct insert was confirmed by restriction digestion and Sanger sequencing.

### Agrobacterium-Mediated Transformation

A modification of the original *M. polymorpha* sporeling *Agrobacterium*-mediated stable transformation method was used to transform the *Marchantia* cam-1/2 spores as described before.^23^

### Auronidin Extraction and Quantification

The pigment was extracted from fresh thallus tissue using methanol: water: formic acid (80:19:1) as described before.^6^ All analyses were done using at least 3 replicates. Approximately 100 mg of thalli were crushed using a plastic pestle in a 1.5 mL tube in the presence of 1 mL of the solvent (at 1:10 material-liquor ratio), vortexed for 10 s, and centrifuged for 2 min at 13,300 rpm. 50 μL of the supernatant were transferred to a 384-well polypropylene microplate (Greiner #781209) to be analyzed in a BMG ClariostarPlus plate reader. For fluorescence quantification, excitation at 490 ± 15 nm and emission 570–8 nm (gain: 1512) using the fluorescence intensity end-point mode and normalized with respect to fresh weight. Sample values were collected as 1 mm orbital average and adjusted by subtracting the fluorescence values of the blank. To optimize the emission and excitation parameters we followed the literature^3^ and measured the absorbance spectra (350–650 nm) using the absorbance mode, and the emission using the fluorescence intensity emission spectrum mode, collecting from 549 to 660 nm with excitation at 490 ± 15 nm. Serial dilutions were performed to confirm the linear relation of the signal (Supplemental Figure 1).

### Dyeing Fibers

Cotton fibers were dyed using an auronidin *M. polymorpha* pigment extract by a methanol solution as described before but using a material-liquor ratio of 1:4. We adapted a protocol for exhaustion dyeing method described before for Sorghum husk.^24^ Briefly, commercial cotton yarn was first scoured in water with soup for 40 min and then incubated with the extract at 30% weight of fiber ratio at room temperature until the solvent was completely evaporated overnight. Gallnut and aluminum acetate (Wonkyweaver) following manufacturing instruction at 10% weight of fiber ratio. Fastness was tested using distilled wated at 50 °C for 30 min and dried.

### Data Analysis

To obtain plots and statistical analysis, data were processed using R v 4.4.1 software and RStudio. Statistical analyses were done using the built-in statistics package using pairwise *t* tests comparing each time to the corresponding control and corrected by Bonferroni using default parameters. Plots were made by using the ggpubr package. Pictures were taken using either a Keyence VHX-5000 digital microscope or a Leica M205 FA stereo microscope equipped with a YFP fluorescence filter and analyzed using ImageJ/Fiji. The thallus area, the exposed area, was quantified from pictures with ImageJ/Fiji software with 3 biological replicates.

## Supplementary Material





## Notes

Supplemental Figure 1 showing serial dilution regression analysis of plant extract fluorescence; Supplemental Figure 2 showing relative thallus area of different inducible transgenic lines (PDF)
Supplemental Table 1 listing vectors and parts used for cloning (XLSX)
