# Supplementary material for: Engineering Auronidin Production in Marchantia polymorpha Enables a New Class of Plant-Derived Textile Pigments
Source: ACS Synth Biol. 2026 Jul 18;15(8):3139–45. doi: 10.1021/acssynbio.6c00118 (PMC13505330; doi:10.1021/acssynbio.6c00118)
Supplement: Supplementary file 1 [file sb6c00118_si_001.pdf]

Engineering auronidin production in *Marchantia polymorpha* enables a new class of plant-derived textile pigments

Aurore Lormet[1], Jim Haseloff[1], Facundo Romani[1]*

[1] Department of Plant Sciences, University of Cambridge, Cambridge, UK.

* Author for correspondence: fr391@cam.ac.uk

SUPPORTING INFORMATION.

Supplemental Figure 1. Serial dilution regression analysis shows linear fluorescence relationship.

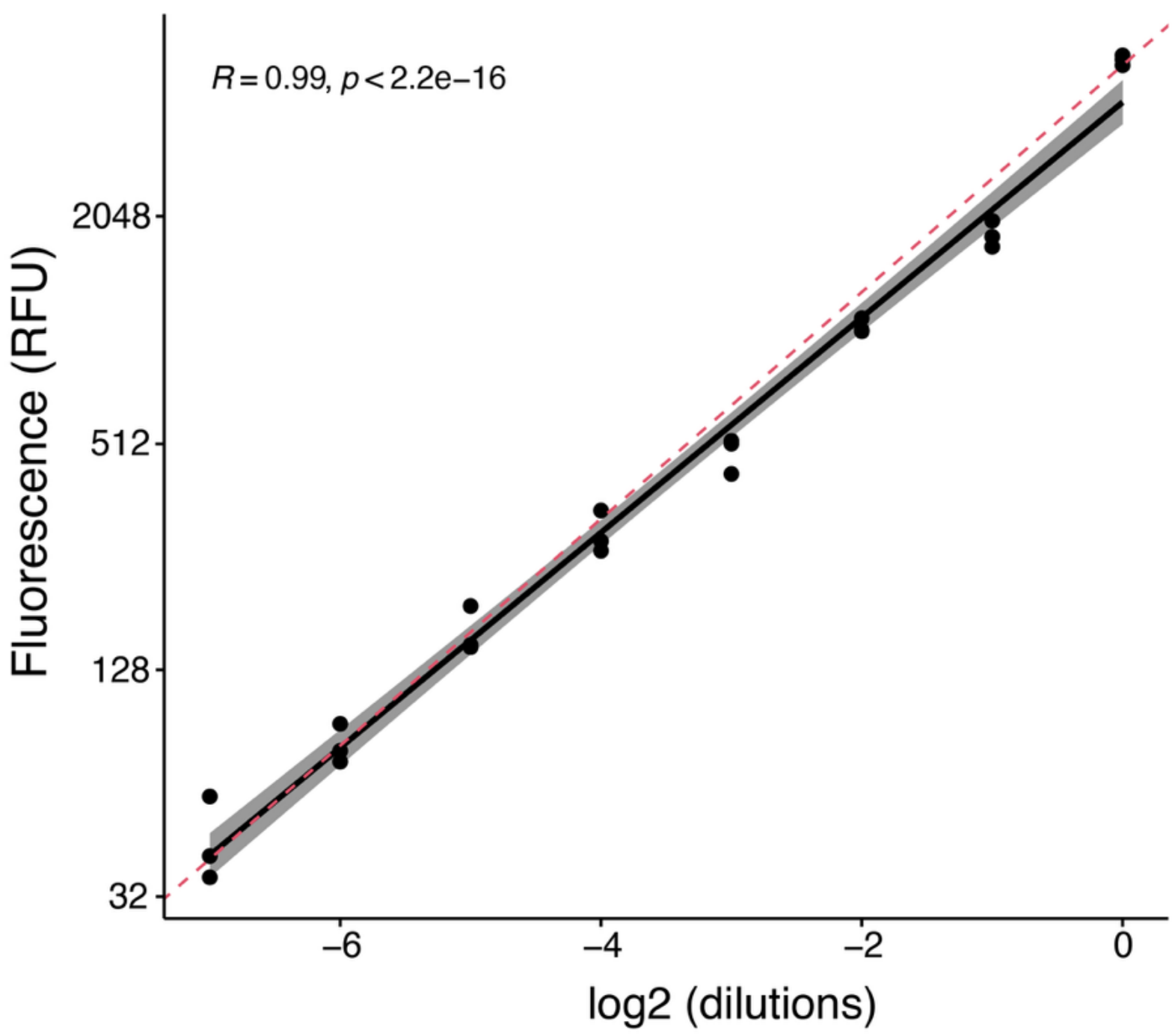

Supplemental Figure 2. Relative thallus size of the 5 different inducible line plants (14 days old). Each bar represents an independent transformant for that promoter construct and the mean of three biological replicates with an error bar for standard deviation between the replicates. Letters above the bars indicate statistically significant differences between means (Anova and Kruskal test at P<0,05 respectively).

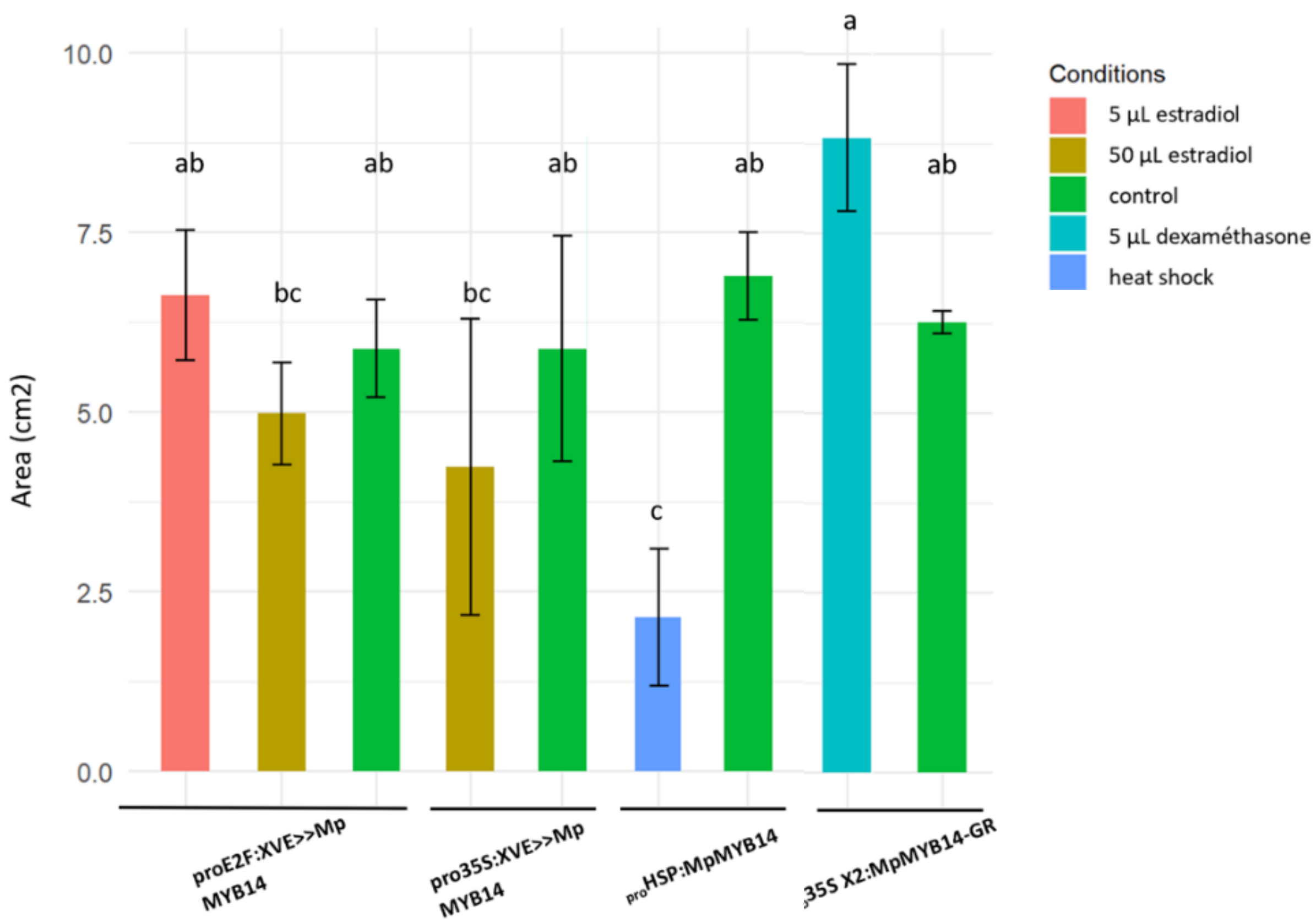

Supplementary Table 1. Vectors and parts used for cloning.
